# NCS‐1 expression is higher in basal breast cancers and regulates calcium influx and cytotoxic responses to doxorubicin

**DOI:** 10.1002/1878-0261.12589

**Published:** 2019-11-11

**Authors:** Alice H. L. Bong, Mélanie Robitaille, Michael J. G. Milevskiy, Sarah J. Roberts‐Thomson, Gregory R. Monteith

**Affiliations:** ^1^ School of Pharmacy The University of Queensland Brisbane Qld Australia; ^2^ ACRF Stem Cells and Cancer Division The Walter and Eliza Hall Institute of Medical Research Parkville Vic. Australia; ^3^ Mater Research Institute Translational Research Institute The University of Queensland Brisbane Qld Australia

**Keywords:** basal breast cancer, calcium, chemotherapy, NCS‐1, ORAI1

## Abstract

Neuronal calcium sensor‐1 (NCS‐1) is a positive modulator of IP_3_ receptors and was recently associated with poorer survival in breast cancers. However, the association between NCS‐1 and breast cancer molecular subtypes and the effects of NCS‐1 silencing on calcium (Ca^2+^) signaling in breast cancer cells remain unexplored. Herein, we report for the first time an increased expression of NCS‐1 in breast cancers of the basal molecular subtype, a subtype associated with poor prognosis. Using MDA‐MB‐231 basal breast cancer cells expressing the GCaMP6m Ca^2+^ indicator, we showed that NCS‐1 silencing did not result in major changes in cytosolic free Ca^2+^ increases as a result of endoplasmic reticulum Ca^2+^ store mobilization. However, NCS‐1 silencing suppressed unstimulated basal Ca^2+^ influx. NCS‐1 silencing in MDA‐MB‐231 cells also promoted necrotic cell death induced by the chemotherapeutic drug doxorubicin (1 µm). The effect of NCS‐1 silencing on cell death was phenocopied by silencing of ORAI1, a Ca^2+^ store‐operated Ca^2+^ channel that maintains Ca^2+^ levels in the endoplasmic reticulum Ca^2+^ store and whose expression was significantly positively correlated with NCS‐1 in clinical breast cancer samples. This newly identified association between NCS‐1 and basal breast cancers, together with the identification of the role of NCS‐1 in the regulation of the effects of doxorubicin in MDA‐MB‐231 breast cancer cells, suggests that NCS‐1 and/or pathways regulated by NCS‐1 may be important in the treatment of basal breast cancers in women.

## Graphical abstract

Neuronal calcium sensor‐1 (NCS‐1) expression is higher in basal breast cancers. Silencing of NCS‐1 suppresses basal calcium (Ca^2+^) influx and increases cell death induced by doxorubicin treatment in basal breast cancer cells. NCS‐1 expression positively correlates with the store‐operated Ca^2+^ channel, Orai1. This work provides further insight into the role of NCS‐1 in modulating therapeutic responses in basal breast cancer.

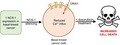

**Abbreviations**
Ca^2+^: calcium
ER: endoplasmic reticulum
IP3: inositol triphosphate
NCS‐1: neuronal calcium sensor‐1
TCGA: The Cancer Genome Atlas
TRPV6: transient receptor potential vanilloid 6

## 1 Introduction

Aberrations in calcium (Ca^2+^) signaling and associated regulatory proteins such as Ca^2+^ channels occur in a variety of cancers (Stewart *et al.*, 2015). Cancer cells may remodel their intracellular Ca^2+^ signaling machinery to favor tumorigenic processes that enable continued survival and proliferation (Prevarskaya *et al.*, 2014). For example, prostate cancers exhibit increased expression of Ca^2+^ channels such as transient receptor potential vanilloid 6 (TRPV6) (Fixemer *et al.*, 2003) and ORAI3 channels (Dubois *et al.*, 2014) compared to normal prostate tissues. The remodeling of ORAI3 channel expression in cancer cells causes a shift in Ca^2+^ influx from a store‐regulated mechanism normally used by healthy prostate cells toward a store‐independent one that confers apoptotic resistance and proliferative signaling (Dubois *et al.*, 2014). Specific remodeling of Ca^2+^ signaling is evident during tumor progression and is also seen between different cancer subtypes such as in breast cancer. Expression of the ORAI1 Ca^2+^ influx channel is higher in breast cancers of the basal molecular subtype, which are often triple‐negative (i.e., do not express estrogen, progesterone, and HER2 receptors) compared to nonbasal breast cancers (McAndrew *et al.*, 2011). Conversely, luminal breast cancer cell lines generally have higher ORAI3 expression and exhibit greater ORAI3‐mediated Ca^2+^ entry compared to basal breast cancer cell lines (Motiani *et al.*, 2010; Motiani *et al.*, 2013).

Studies of Ca^2+^ signaling in cancer cells have usually focused on plasma membrane Ca^2+^ permeable ion channels and their regulators (Deliot and Constantin, 2015; Vashisht *et al.*, 2015). In contrast, far fewer studies have assessed the potential remodeling of proteins regulating endoplasmic reticulum (ER) Ca^2+^ homeostasis in cancer. The ER is the main intracellular store for releasable Ca^2+^ in response to activating stimuli. The major proteins regulating ER Ca^2+^ homeostasis in epithelial cells are the sarco/ER Ca^2+^ ATPase (SERCA) pumps, which actively sequester cytosolic Ca^2+^ into the ER lumen, and inositol triphosphate receptors (IP3R), which release ER Ca^2+^ in response to IP_3_‐mobilizing agonists such as ATP. The release of ER Ca^2+^ through IP3R activates an ER‐refilling mechanism called store‐operated Ca^2+^ entry (SOCE). During SOCE, an ER‐resident Ca^2+^ sensor protein called stromal interaction molecule 1 (STIM1) senses the reduced ER Ca^2+^ levels and oligomerizes to form a Ca^2+^‐permeant protein complex with ORAI channels on the plasma membrane, facilitating Ca^2+^ influx. Dysregulation in ER Ca^2+^ homeostasis is associated with some cancerous phenotypes including the ability to resist apoptosis and the promotion of prosurvival signaling, which can influence response to anticancer therapies (Pedriali *et al.*, 2017). Abnormal SERCA expression and/or activity is associated with some cancers including colorectal (Fan *et al.*, 2014; Yang *et al.*, 2015) and blood cancers (Roti *et al.*, 2013). These alterations could be targeted for cancer treatment. Indeed, mipsagargin, a prostate‐specific membrane antigen‐based prodrug targeting the SERCA pumps, is currently in clinical trials for the treatment of solid tumors (Mahalingam *et al.*, 2016). Alterations in ER Ca^2+^ signaling can also be mediated through protooncogenes such as the Bcl‐2 family of antiapoptotic proteins, which lower ER Ca^2+^ levels by increasing ER Ca^2+^ ‘leak’ (Bittremieux *et al.*, 2016; Foyouzi‐Youssefi *et al.*, 2000). Apart from these more well‐defined mechanisms of ER Ca^2+^ store regulation, there are also proteins which indirectly modulate ER Ca^2+^ signals such as the neuronal calcium sensor‐1 (NCS‐1).

Neuronal calcium sensor‐1 is a 22‐kDa high‐affinity EF‐hand‐containing Ca^2+^ sensor protein with structural similarity to calmodulin. NCS‐1 contains an N‐terminal myristoyl group that allows Ca^2+^‐dependent binding to proteins and cellular membranes. NCS‐1 interacts with IP3R and increases its opening probability (Schlecker *et al.*, 2006), and NCS‐1 silencing reduces IP_3_‐mediated Ca^2+^ signals mediated by ATP in neuroblastoma cells (Boehmerle *et al.*, 2007) and endothelin in cardiomyocytes (Zhang *et al.*, 2010). NCS‐1 is widely expressed in adult neuronal cells (Nakamura and Wakabayashi, 2012; Weiss *et al.*, 2010) and is involved in the regulation of neurotransmission activity important for learning, memory, and synaptic plasticity (Sippy *et al.*, 2003; Weiss *et al.*, 2010). NCS‐1 also regulates Akt‐dependent prosurvival signaling in neurons (Nakamura *et al.*, 2006) and cardiomyocytes (Nakamura *et al.*, 2016). NCS‐1 is implicated in a variety of disease states such as schizophrenia and bipolar disorder (Boeckel and Ehrlich, 2018; Koh *et al.*, 2003). In the context of cancer, NCS‐1 is a potential target for the prevention of paclitaxel‐induced peripheral neuropathy (Mo *et al.*, 2012). Ehrlich *et al.* showed that paclitaxel treatment enhances the binding of NCS‐1 to IP3R in neuronal cells (Boehmerle *et al.*, 2006) and prolonged paclitaxel treatment results in dysregulation of IP_3_‐dependent Ca^2+^ signaling due to the degradation of NCS‐1 via Ca^2+^‐mediated calpain activation (Boehmerle *et al.*, 2007). This dysregulated Ca^2+^ signaling was proposed to cause the peripheral neuropathy induced by paclitaxel. More recently, NCS‐1 was shown to play a role in breast cancer invasion and migration *in vitro*, and higher NCS‐1 expression correlates with poorer survival in breast cancer patients (Moore *et al.*, 2017).

Despite the involvement of NCS‐1 in regulating Ca^2+^ homeostasis and its association with breast cancer, no studies have assessed the role of NCS‐1 in intracellular Ca^2+^ signaling in breast cancer cells. The association of NCS‐1 expression with breast cancer molecular subtypes also remains unexplored. Here, we report for the first time that NCS‐1 expression is increased in the basal breast cancer molecular subtype. We also demonstrate that siRNA‐mediated silencing of NCS‐1 attenuated unstimulated basal Ca^2+^ influx in basal breast cancer cells. Silencing NCS‐1 enhanced doxorubicin‐induced breast cancer cell death, which was a phenomenon phenocopied by the silencing of the Ca^2+^ store refilling channel ORAI1. These studies highlight a potentially important role for NCS‐1 in the regulation of Ca^2+^ influx pathways important in the induction of cell death by some therapies in basal breast cancer cells.

## 2 Materials and methods

### 2.1 Assessment of NCS‐1 expression in breast cancer molecular subtypes

Expression data for NCS‐1 in breast tumors were downloaded as log2‐RSEM values from The Cancer Genome Atlas [TCGA; (Cancer Genome Atlas, 2012)] patient database and stratified into the molecular subtypes: Luminal A (*n* = 409), Luminal B (*n* = 189), HER2 (*n* = 67), Basal (*n* = 132), and Normal‐like (*n* = 22) (Cancer Genome Atlas, 2012). The gene expression of NCS‐1 in breast cancer cell lines categorized into Luminal, HER2‐amplified and Basal (Neve *et al.*, 2006) molecular subtypes was assessed using publicly available microarray data from Array Express (accession number: E‐MTAB‐181) (Heiser *et al.*, 2012).

A gene expression heatmap was generated using gene expression data from the TCGA database. The TCGA gene expression data were mean‐centered and hierarchically clustered using Multiple Experiment Viewer (v4.8.1; Saeed *et al.*, 2003) with Manhattan‐based average‐linkage clustering. Displayed above the heatmap are the PAM50 molecular subtypes. Molecular markers typical of the different molecular subtypes were used in the clustering.

### 2.2 Patient survival analysis in basal breast cancer

Overall patient survival in basal breast cancers based on NCS‐1 expression was assessed using the Kaplan–Meier Plotter web‐based tool (Gyorffy *et al.*, 2010). Affymetrix probe IDs used in the analyses were the mean of 230146_s_at, 222570_at, and 238753_at, and high or low NCS‐1 expression was stratified using the ‘auto‐select best cutoff’ function. This function assesses median, tertile, and quartile expression cutoffs and utilizes the best cutoff for the given gene and dataset. Hazard ratio and log‐rank *P* values are shown in the figure.

### 2.3 Gene correlation analysis

Gene correlation analyses were performed on the R2 Genomics Visualization Platform (http://r2.amc.nl) using TCGA microarray datasets. Correlation coefficients between NCS‐1 and assessed genes are shown as *R*‐values and significance of correlations is shown as *P*‐values.

### 2.4 Cell culture

GCaMP6m‐expressing MDA‐MB‐231 (GCaMP6m‐MDA‐MB‐231) cells were developed as previously described (Bassett *et al.*, 2018). Parental MDA‐MB‐231 basal breast cancer cells were obtained from American Type Culture Collection. Cells were cultured and passaged in Dulbecco’s Modified Eagle’s Medium (DMEM; Sigma‐Aldrich, Castle Hill, NSW, Australia) supplemented with 10% FBS (HyClone, GE Life Sciences, Marlborough, MA, USA), 4 mm l‐glutamine, and 400 µg·mL^−1^ hygromycin (Invitrogen, Carlsbad, CA, USA). Cell line authentication was performed with STR profiling at the QIMR Berghofer Institute, Brisbane. Mycoplasma testing was done biannually using the Lonza MycoAlert™ (Basel, Switzerland) Mycoplasma Detection Kit.

### 2.5 NCS‐1 lentiviral production and transduction

Human NCS‐1 was amplified from Applied Biological Material (LV800666) plasmid by PCR and cloned into the pCDH‐EF1‐FHC lentiviral vector (Addgene # 64874). Lentiviral particles were produced in HEK293T cells with second‐generation packaging plasmids and Lipofectamine 3000 transfection. The medium containing viral particles was collected after 48 h. GCaMP6m‐MDA‐MB‐231 cells were subsequently transduced with the empty vector (EV) or human NCS‐1 in the presence of polybrene (8 µg·mL^−1^). The viral media were replaced with fresh media 24 h after infection and cells were selected with puromycin (2 µg·mL^−1^) 48 h after viral infection.

### 2.6 siRNA transfection

Cells were seeded into 96‐well plates (4 × 10^3^ cells per well) in antibiotic‐free complete media 24 h before siRNA transfection. For siRNA transfection, cells were incubated in 8% FBS transfection media containing DharmaFECT4 (0.1 µL per well; Dharmacon, Horizon Discovery, Cambridge, UK) and 100 nm of either SMARTpool ON‐TARGETplus NCS‐1 siRNA (L‐013024‐01‐0005; Dharmacon, Horizon Discovery) or ON‐TARGETplus Nontargeting (NT) Control siRNAs (D‐001810‐10‐01; Dharmacon, Horizon Discovery) at a final concentration of 100 nm per well. Cells were transfected with siRNAs for 96 h prior to Ca^2+^‐imaging experiments and for 24 h prior to doxorubicin or paclitaxel treatments.

### 2.7 RNA isolation and real‐time PCR

Total RNA was isolated from cells and purified using the RNeasy Mini Kit (Qiagen, Hilden, Germany). RNA concentrations were determined using a NanoDrop 2000 UV‐Vis Spectrophotometer (Thermo Fisher, Waltham, MA, USA). RNA was reverse transcribed into cDNA using the Omniscript Reverse Transcription Kit (Qiagen). cDNA amplification was performed using the TaqMan Fast Universal PCR Master Mix (Applied Biosystems, Foster City, CA, USA). Real‐time PCR reactions were performed using the StepOne Plus Real‐Time PCR System (Applied Biosystems) under universal cycling conditions. TaqMan Gene Expression Assays (Applied Biosystems) used were: NCS‐1 (Hs00179522_m1), ORAI1 (Hs03046013_m1), ITPR1 (Hs00181881_m1), ITPR2 (Hs00181916_m1), ITPR3 (Hs01573555_m1), SERCA1 (Hs01092295_m1), SERCA2 (Hs00544877_m1), and SERCA3 (Hs00193090_m1). Relative gene expression was quantitated using the comparative C*_T_* method (ΔΔC*_T_*), normalized to 18s rRNA (4310893E; Applied Biosystems).

### 2.8 Immunoblotting

Cells were lysed using cold protein lysis buffer containing protease and phosphatase inhibitors (Roche Applied Science, Penzberg, Germany). Gel electrophoresis was performed using Mini‐PROTEAN® TGX Pre‐cast Gels and protein was transferred to a poly(vinylidene difluoride) membrane (Bio‐Rad Laboratories, Hercules, CA, USA). Blots were blocked for 1 h in 5% skim milk in phosphate‐buffered saline containing 0.1% Tween‐20 (PBST) before incubating with the following primary antibodies: NCS‐1 (diluted 1 : 500, 129166; Abcam, Cambridge, UK), ORAI1 (diluted 1 : 1000, 4281, ProSci Inc., Poway, CA, USA), PARP‐1 (diluted 1 : 1000, 9542; Cell Signaling, Beverly, MA, USA), and β‐actin (diluted 1 : 10 000, A5441; Sigma). All primary antibodies were diluted using 5% skim milk in PBST and incubated overnight at 4 °C except β‐actin, which was incubated for 1 h at room temperature. Goat anti‐mouse (170‐6516) and goat anti‐rabbit (170‐6515) horseradish peroxidase conjugate secondary antibodies were diluted 1 : 10 000 and incubated for 1 h at room temperature. Protein bands were imaged using the SuperSignal West Dura Extended Duration Chemiluminescent Substrate (Thermo Fisher Scientific) on the Bio‐Rad ChemiDoc Imaging System (Bio‐Rad Laboratories). β‐Actin was used as the loading control. Quantification of protein band density was performed using the Bio‐Rad imagelab software (version 5.2.1) as per user guidelines.

### 2.9 Calcium imaging using fluorometric imaging plate reader (FLIPR)

Cells were seeded into black‐walled 96‐well plates (Corning Incorporated, Corning, NY, USA) at a density of 4 × 10^3^ cells per well and Ca^2+^ imaging was performed 96 h post‐siRNA transfection using the FLIPR^TETRA^ (Molecular Devices, San Jose, CA, USA). GCaMP6m‐MDA‐MB‐231 cells with exogenous overexpression of NCS‐1 or expressing the EV control were seeded at a density of 1 × 10^4^ cells per well, and Ca^2+^ imaging was performed 72 h after seeding. Briefly, media was removed from the wells, washed once, and then replaced with physiological salt solution (PSS, composed of 10 mm HEPES, 5.9 mm KCl, 1.4 mm MgCl_2_, 1.2 mm NaH_2_PO_4_, 5 mm NaHCO_3_, 140 mm NaCl, 11.5 mm glucose, pH 7.2) containing nominal Ca^2+^ (no added CaCl_2_) and incubated for 15 min at room temperature prior to Ca^2+^‐imaging studies. For the assessment of ER Ca^2+^ signaling, IP_3_‐mobilizing agents (ATP; Sigma‐Aldrich) at 1, 3, and 100 µm and trypsin (Sigma‐Aldrich) at 1, 10, and 100 nm concentrations) and cyclopiazonic acid (CPA; Sigma at 10 µm concentration) prepared in PSS were added to wells. To assess unstimulated Ca^2+^ influx, 1.8 mm CaCl_2_ was added. A 100 µm concentration of bis(2‐aminophenoxy)ethane tetraacetic acid (BAPTA; Invitrogen™) was included during the addition of reagents. For assessment of SOCE, BAPTA (100 µm) was first added to cells preincubated for 15 min in PSS nominal, followed by CPA (10 µm) addition to mediate ER store depletion. CaCl_2_ (1.8 mm) was then added after 700 s to facilitate SOCE. An ORAI1 inhibitor Synta66 (10 µm; Sigma) was included in experiments assessing basal Ca^2+^ influx and SOCE. Changes in fluorescence intensity relative to baseline fluorescence over time were expressed as cytosolic Ca^2+^ changes (ΔF/F_o_) and were measured at 470–495 nm excitation and 515–575 nm emission wavelengths and analyzed using the screenworks Software (Molecular Devices).

### 2.10 Assessment of cell proliferation and death using epifluorescence microscopy

Cell proliferation was assessed using the Click‐iT™ EdU Alexa Fluor™ 555 imaging kit (Invitrogen). Briefly, 24 and 48 h after siRNA transfection and doxorubicin (24 h) treatments, cells were incubated with EdU (10 µm) for 1 h at 37 °C. Cells were then fixed with 4% paraformaldehyde for 15 min, washed with PBS containing 3% BSA, and permeabilized using 0.5% Triton‐X for 20 min. Cells were then incubated in the dark with the Click‐iT reaction cocktail (containing Alexa Fluor azide) for 1 h, prepared according to manufacturer’s instructions. Cell nuclei were stained using Hoecsht 33342 (Invitrogen, Vista, CA, USA; 400 nm).

For the assessment of necrotic cell death, at 24 h post‐siRNA transfection, medium was replaced with 10% FBS and HEPES‐buffered Fluorobrite™ DMEM (Invitrogen) or Fluorobrite media containing 0.03 or 1 µm doxorubicin (Merck Millipore, MilliporeSigma, Burlington, MA, USA) and incubated for 24 h before replacing with drug‐free Fluorobrite DMEM. After 72 h, cells were stained with Hoechst 33342 (10 µg·mL^−1^; Invitrogen) and propidium iodide (PI; 1 µg·mL^−1^; Invitrogen). Cells for both proliferation and death experiments were imaged using the ImageXpress Micro (Molecular Devices) epifluorescence microscope, using the DAPI and Cy3 filter sets to assess EdU‐positive cells or PI‐positive cells as described previously (Curry *et al.*, 2012; Peters *et al.*, 2012). The multiwavelength cell scoring module (MetaXpress 6.0) was used to assess the percentage of EdU‐positive or PI‐positive cells.

### 2.11 Statistical analysis

Statistical analyses of individual experiments were performed using graphpad prism (version 7; GraphPad Software, San Diego, CA, USA) as described in the corresponding figure legends. Data are presented as mean ± SEM (of three independent experiments).

## 3 Results

### 3.1 NCS‐1 expression is higher and is predictive of poorer survival in the basal breast cancer molecular subtype

**Figure 1:**
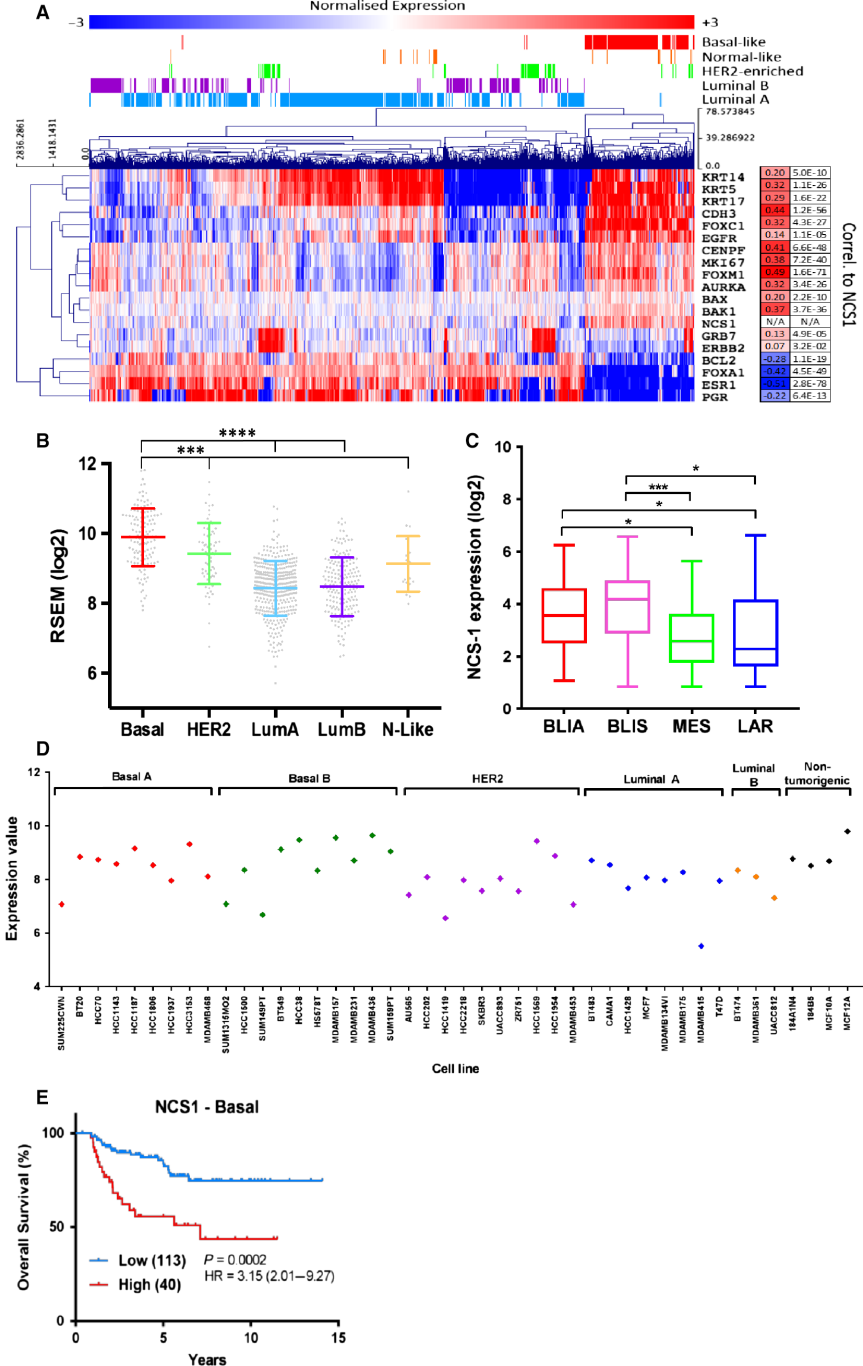
NCS‐1 expression is correlated with the basal breast cancer molecular subtype. (A) Clustered heatmap of normalized RNA‐Seq expression data, where low expression is indicated in blue and high expression in red. Data are log2 mean‐centered RSEM values sourced from TCGA. Displayed on the right are the Pearson’s correlation coefficients of NCS1 expression versus the molecular markers, and corresponding *P*‐values are indicated. Indicated above the heatmap are the PAM50 molecular subtypes for each breast tumor. (B) Relative gene expression of NCS‐1 in breast tumors from TCGA database (Cancer Genome Atlas, 2012) categorized according to breast cancer molecular subtypes. Statistical analysis was performed using a one‐way ANOVA with Tukey’s test. ****P < 0.0001, ***P < 0.0002. (C) Relative gene expression levels of NCS‐1 in TNBC subtypes as described by Burstein et al. (2015). NCS‐1 expression is higher in BLIA and BLIS compared to MES and LAR subtypes. Data were downloaded from R2 Genomics Analysis Platform and plotted in graphpad prism. Statistical analysis was performed using a one‐way ANOVA with Tukey’s test. ***P < 0.001, * *P* < 0.05. (D) NCS‐1 expression in 40 breast cancer cell lines grouped to the molecular subtypes and in 4 nontumorigenic, breast epithelial cell lines. Data obtained from Array Express (accession number E‐MTAB‐181) (Heiser et al., 2012). (E) OS of patients with basal breast cancer stratified to NCS‐1 expression. Data were sourced from the Kaplan–Meier Plotter online tool (Gyorffy et al., 2010)

Given that NCS‐1 was recently reported to be associated with increased breast tumor aggression and poor prognosis (Moore *et al.*, 2017), we explored if NCS‐1 is associated with any of the breast cancer intrinsic molecular subtypes using TCGA breast cancer database (Cancer Genome Atlas, 2012). Hierarchical clustering of NCS‐1 and breast cancer molecular markers showed a positive correlation with basal and proliferative markers, such as KRT5, 14 and 17, CDH3, FOXC1, FOXM1, EGFR, and MKI67 (Fig. 1A). Conversely, NCS‐1 expression is negatively correlated to FOXA1, ESR1, and PGR, which are more commonly associated with the breast cancer luminal subtype. We found that NCS‐1 expression is also significantly higher in the basal molecular subtype (Fig. 1B) compared to other breast cancer subtypes. NCS‐1 levels were also highest in the basal‐like immune‐activated (BLIA) and basal‐like immune‐suppressed (BLIS) subtypes within the triple‐negative breast cancer (TNBC) subtypes (Burstein *et al.*, 2015) (Fig. 1C). We also observed a trend toward increased NCS‐1 expression in basal breast cancer cell lines compared to HER2 and luminal breast cancer cell lines (Fig. 1D).

Finally, to assess if NCS‐1 expression had any association with patient survival in basal breast cancers, we used the Kaplan–Meier Plotter online tool to stratify the overall survival (OS) of breast cancer patients with basal tumors based on NCS‐1 gene expression (Gyorffy *et al.*, 2010). As shown in Fig. 1E, higher NCS‐1 expression correlates with a poorer OS in the basal breast cancer patient subgroup (HR = 3.15, *P* = 0.0002), further implicating the significance of NCS‐1 in basal breast cancers.

### 3.2 NCS‐1 silencing has no major effect on ER Ca^2+^ signaling but suppresses unstimulated, basal Ca^2+^ influx in MDA‐MB‐231 cells

**Figure 2:**
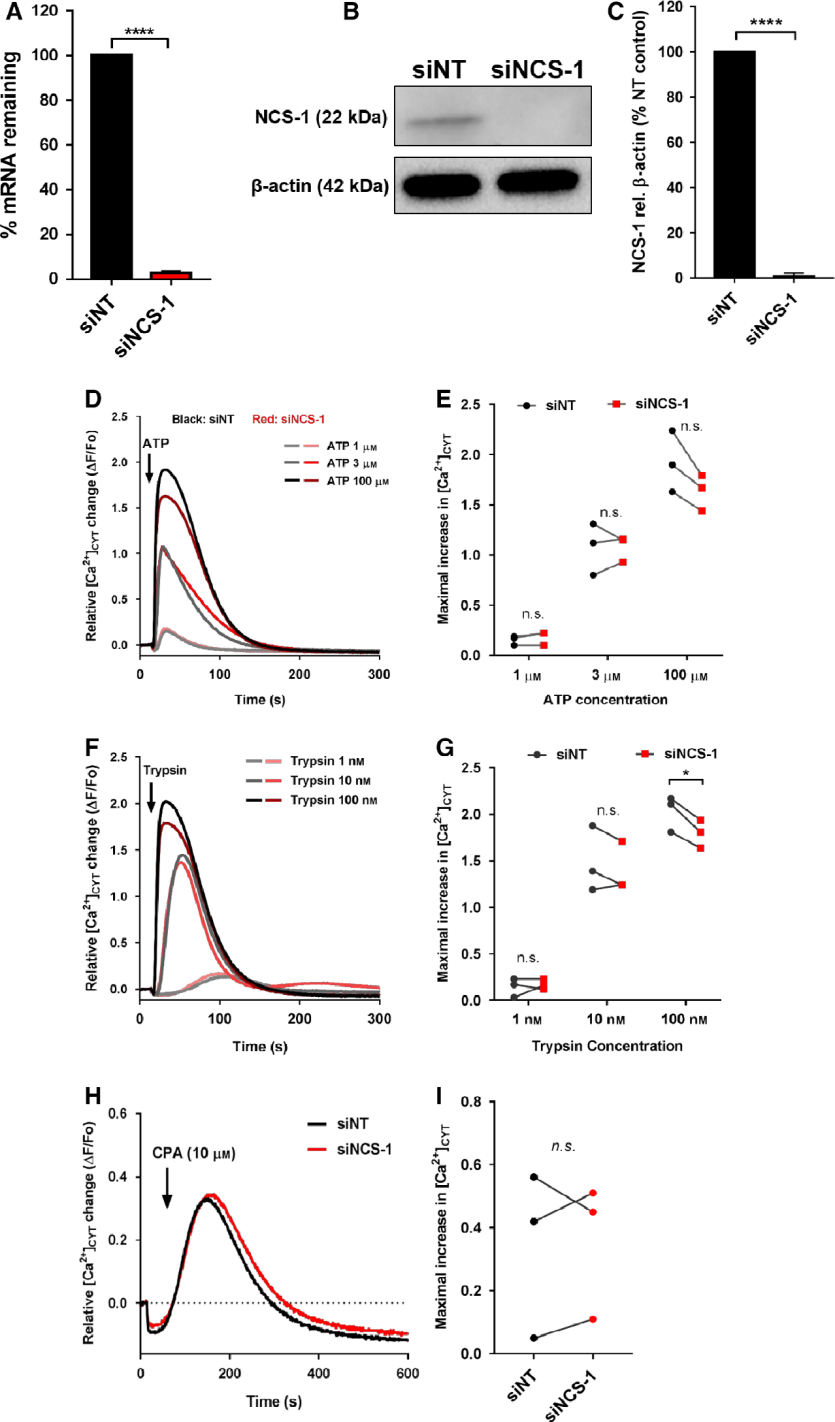
Effect of NCS‐1 silencing on ER Ca^2+^ signaling. (A‐C) Confirmation of NCS‐1 siRNA‐mediated silencing using real‐time RT‐PCR and immunoblotting. Bar graphs show the mean ± SEM of three independent experiments. Traces (D, F, H) show mean relative [Ca^2+^]_CYT_ change mediated by ATP (1, 3, and 100 µm), trypsin (1, 10, and 100 nm), and CPA (10 µm) addition. (E), (G), and (I) show maximal increases in relative [Ca^2+^]_CYT_ levels induced by ATP, trypsin, and CPA, respectively. Data points show the mean of triplicate wells from each biological replicate matching NT siRNA and NCS‐1 siRNA treatment to the same biological replicate. Statistical analysis was performed using *t*‐tests. *P < 0.05; ****P < 0.0001; n.s. is not significant.

Due to the role of NCS‐1 as a positive regulator of IP3Rs (Boehmerle *et al.*, 2007; Schlecker *et al.*, 2006) and the lack of studies defining a role for NCS‐1 in Ca^2+^ signaling in breast cancer cells, we assessed two possible consequences of NCS‐1 silencing in the GCaMP6m‐MDA‐MB‐231 breast cancer cell line. These two potential consequences reduced IP_3_‐mediated Ca^2+^ store release after G‐protein‐coupled receptor activation (Berridge, 2016) and reduced compensatory basal Ca^2+^ influx as a result of less basal ER Ca^2+^ leak from IP3Rs (Mignen *et al.*, 2017). We inhibited NCS‐1 expression using siRNAs (Fig. 2A–C) and assessed relative intracellular [Ca^2+^]_CYT_ increases in response to ATP and trypsin. In the absence of extracellular Ca^2+^, ATP and trypsin addition mobilizes ER Ca^2+^ stores through an IP_3_‐mediated pathway. NCS‐1 silencing had no significant effect on [Ca^2+^]_CYT_ increases mediated by any concentration of ATP (Fig. 2D,E) and trypsin at 1 and 10 nm (Fig. 2F,G) concentrations; however, NCS‐1 silencing modestly suppressed Ca^2+^ signals in response to trypsin at 100 nm (Fig. 2G). [Ca^2+^]_CYT_ increases induced by SERCA inhibition using CPA were also not significantly affected by NCS‐1 silencing (Fig. 2H,I). Collectively, these data suggest that NCS‐1 is not a major regulator of ER Ca^2+^ release in MDA‐MB‐231 breast cancer cells.

**Figure 3:**
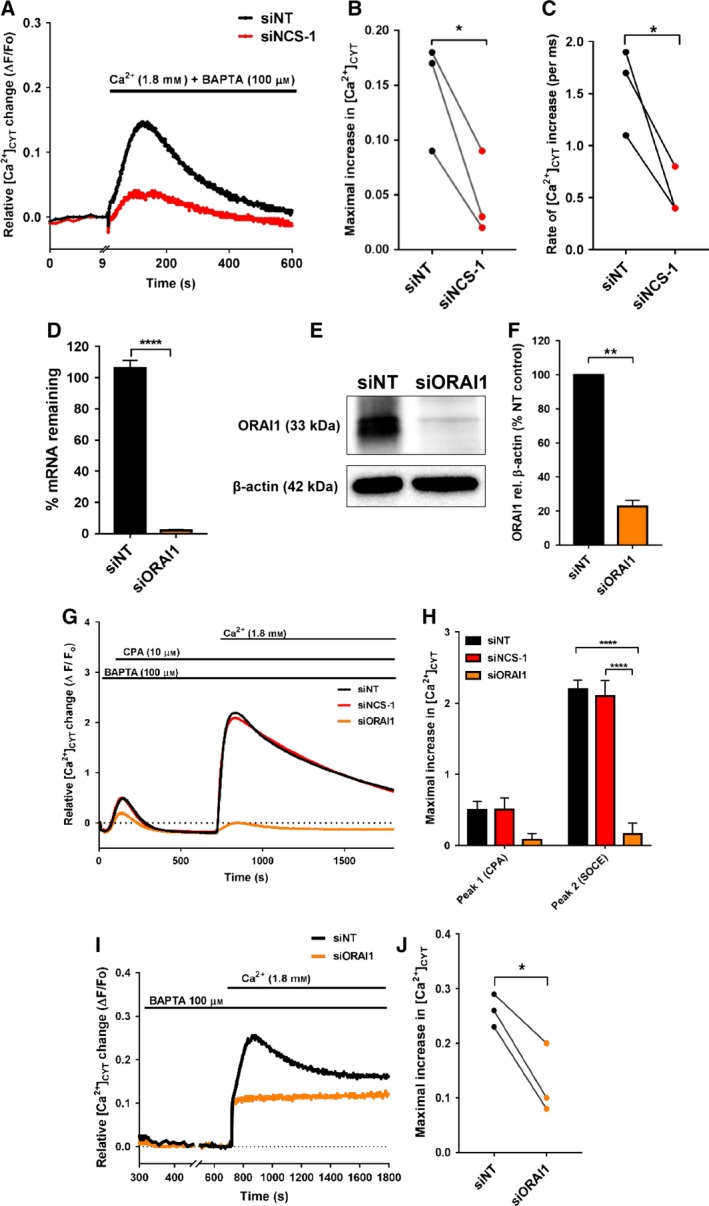
NCS‐1 silencing suppresses unstimulated, basal Ca^2+^ influx which phenocopies ORAI1 silencing. (A) Relative mean [Ca^2+^]_CYT_ increases in GCaMP6m‐MDA‐MB‐231 cells preincubated in PSS nominal and induced by the addition of extracellular Ca^2+^ [CaCl_2_ (1.8 mm) + BAPTA (100 µm)]. Graphs show analyses of (B) maximum relative [Ca^2+^]_CYT_ increase and (C) rate of [Ca^2+^]_CYT_ increase (from 9 to 100 s). (D–F) Confirmation of ORAI1 silencing using real‐time PCR and immunoblotting. (G, H) siRNA‐mediated inhibition of ORAI1 but not NCS‐1 suppresses SOCE, that is, Peak 2 during Ca^2+^ readdition at 750 s. Statistical analysis was performed using a one‐way ANOVA with Bonferroni’s *post‐hoc* test. ****P < 0.001 (I) Trace shows the Ca^2+^ readdition phase of wells not pretreated with CPA from the same experiment shown in (G) from 300 to 1800 s. The trace shows a reduction in unstimulated Ca^2+^ influx as a result of ORAI1 silencing. (J) Bar graph shows the mean ± SEM of the maximal [Ca^2+^]_CYT_ increases during Ca^2+^ readdition from 700 to 1800 s. Statistical analysis was performed using a paired *t*‐test. *P < 0.05; **P < 0.002, ****P < 0.0001.

In some cancer cells, altered Ca^2+^ influx in the absence of external stimuli (unstimulated or basal Ca^2+^ influx) is associated with key tumorigenic traits, such as increased proliferation and migration (Chantome *et al.*, 2013; Feng *et al.*, 2010; Mignen *et al.*, 2017; Peters *et al.*, 2012). Thus, we next investigated the effect of NCS‐1 silencing on unstimulated, basal Ca^2+^ influx. As shown in Fig. 3A, when extracellular Ca^2+^ (1.8 mm) was added to GCaMP6m‐MDA‐MB‐231 cells incubated in nominal Ca^2+^ conditions, the increase in [Ca^2+^]_CYT_ levels induced by the readdition of extracellular Ca^2+^ was attenuated when NCS‐1 was silenced. Analysis of the peak (Fig. 3B) and rate of Ca^2+^ influx from 9 to 100 s (Fig. 3C) revealed a significant decrease in Ca^2+^ influx with NCS‐1 silencing. To assess if NCS‐1 silencing also reduced Ca^2+^ influx through SOCE, we performed a classical SOCE experiment using CPA‐mediated ER Ca^2+^ store depletion, followed by Ca^2+^ readdition. We first confirmed that silencing of the store‐operated Ca^2+^ channel ORAI1 (Fig. 3D–F) eliminated SOCE (Fig. 3G,H). However, silencing of NCS‐1 had no effect on SOCE (Fig. 3G,H). We have previously shown that unstimulated Ca^2+^ influx occurs through ORAI1 in HC11 mammary epithelial cells (Ross *et al.*, 2013). To assess if unstimulated Ca^2+^ influx occurred through ORAI1 in GCaMP6m‐MDA‐MB‐231 cells, we assessed the effect of ORAI1 silencing. As shown in Fig. 3I,J, ORAI1 silencing suppressed unstimulated Ca^2+^ influx. Collectively, these results identify a critical role for NCS‐1 in modulating unstimulated Ca^2+^ influx likely through ORAI1 channels, since ORAI1 silencing phenocopied the effect of NCS‐1 silencing in GCaMP6m‐MDA‐MB‐231 breast cancer cells.

### 3.3 NCS‐1 overexpression reduces ATP‐induced Ca^2+^ release but does not affect unstimulated Ca^2+^ influx

**Figure 4:**
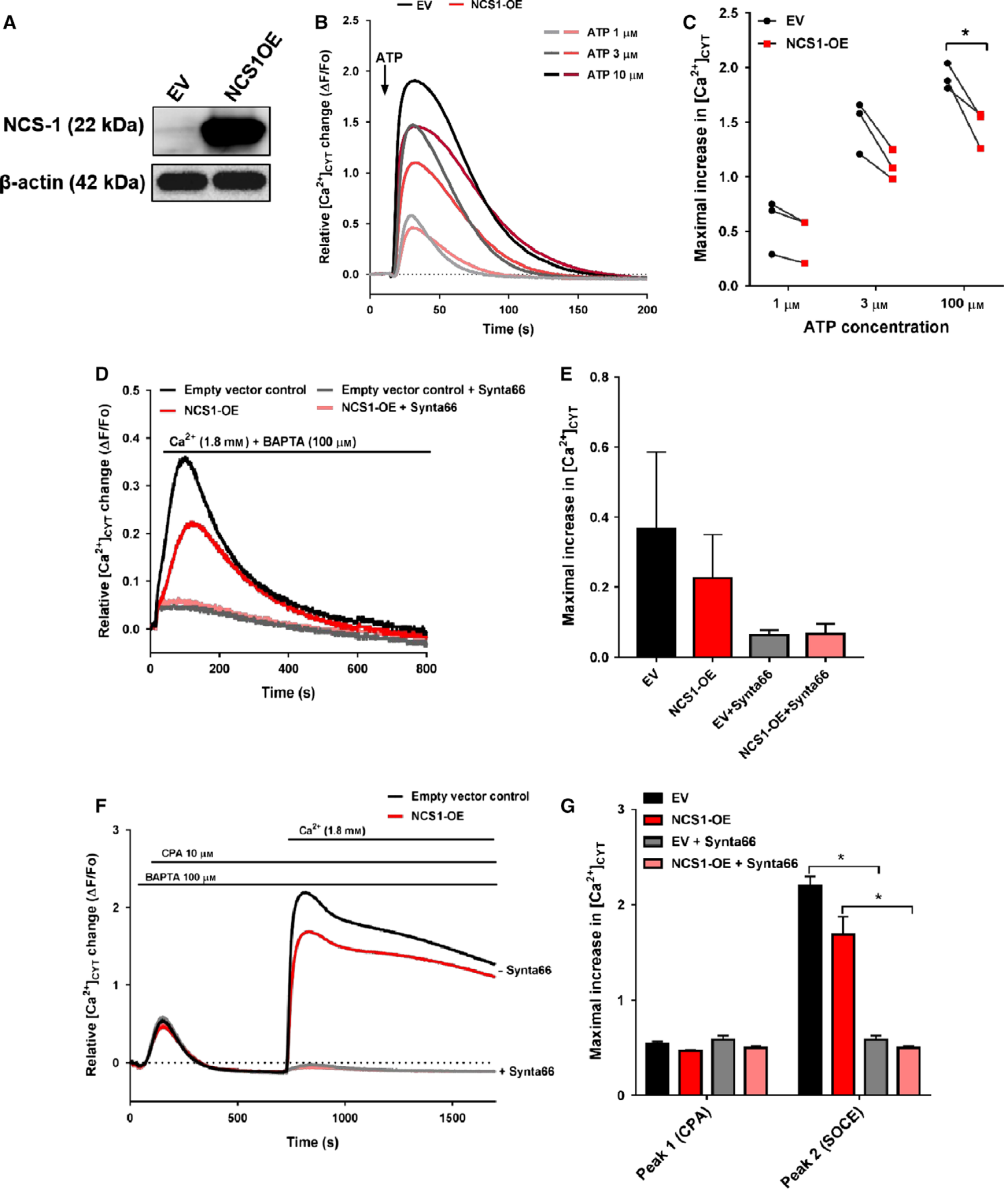
NCS‐1 overexpression reduces ATP‐induced ER Ca^2+^ signals without significant effects on unstimulated Ca^2+^ influx and SOCE. (A) Representative immunoblot showing expression of NCS‐1 in GCaMP6m‐MDA‐MB‐231 cells transduced with EV control or an NCS‐1 lentiviral plasmid (NCS1‐OE), using β‐actin as a loading control. (B) Representative Ca^2+^ trace comparing ATP‐induced ER Ca^2+^ release in EV (black) and NCS1‐overexpressing (red) cells. (C) Graph shows the maximal increase in relative [Ca^2+^]_CYT_ levels induced by 1, 3, and 100 µm ATP, respectively. Data points show the mean of triplicate wells of each biological replicate matching EV cells to NCS1‐overexpressing cells from three independent experiments. Statistical analysis was performed using multiple *t*‐tests. *P < 0.05 (D) Trace shows the mean relative [Ca^2+^]_CYT_ increases as a result of unstimulated Ca^2+^ influx of three independent experiments and the effect of Synta66 addition on EV or NCS1‐overexpressing cells. (E) Bar graph shows mean ± SEM of maximal [Ca^2+^]_CYT_ increases as a result of unstimulated Ca^2+^ influx from three independent experiments. (F) Representative Ca^2+^ trace shows the mean relative [Ca^2+^]_CYT_ increases as a result of SOCE in EV or NCS1‐OE GCaMP6m‐MDA‐MB‐231 cells and the effect of Synta66 addition. (G) Bar graph shows mean ± SEM of peak [Ca^2+^]_CYT_ increases from three independent experiments. Statistical analysis was performed using a one‐way ANOVA with Bonferroni’s *post‐hoc* test. *P < 0.05.

In light of the observed role of NCS‐1 silencing on unstimulated Ca^2+^ influx, we further investigated if this Ca^2+^ influx pathway could be enhanced with NCS‐1 overexpression. We generated stable NCS‐1‐overexpressing GCaMP6m‐MDA‐MB‐231 cells (NCS1‐OE) using lentiviral transduction with a commercially available human NCS‐1 plasmid (Fig. 4A). We first assessed the functional role of NCS1‐OE cells in IP_3_‐mediated ER Ca^2+^ release using ATP, and showed that NCS1‐OE cells reduced ER Ca^2+^ release in response to 100 µm ATP (Fig. 4B,C) compared to GCaMP6m‐MDA‐MB‐231 cells expressing the EV control. We then assessed unstimulated Ca^2+^ influx in NCS1‐OE cells compared to EV cells. As shown in Fig. 4D,E, NCS‐1 overexpression did not enhance unstimulated Ca^2+^ influx in GCaMP6m‐MDA‐MB‐231 cells. Unstimulated Ca^2+^ influx was inhibited with the addition of the ORAI1 inhibitor, Synta66 (Fig. 4D,E). NCS‐1 overexpression also did not have any significant effect on SOCE (Fig. 4F,G). Collectively, these results demonstrate that NCS‐1 is not a major direct regulator of SOCE and that promotion of unstimulated Ca^2+^ influx may already be maximal in GCaMP6m‐MDA‐MB‐231 breast cancer cells.

### 3.4 Correlation between NCS‐1 expression and Ca^2+^ channels or pumps

**Figure 5:**
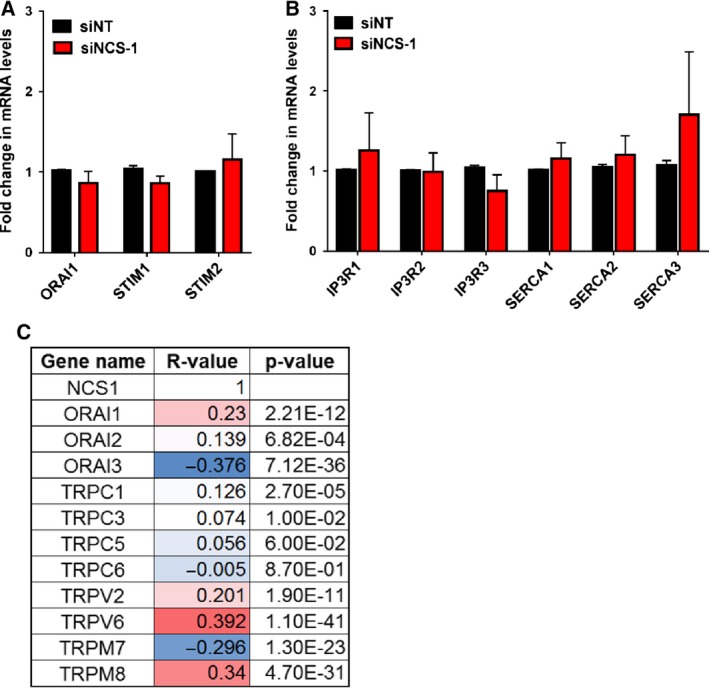
Correlation between expression of NCS‐1 and specific Ca^2+^ regulators. Bar graphs show the fold change in mRNA levels of (A) ORAI1, STIM1, and STIM2 and (B) IP3R and SERCA isoforms 48 h after NCS‐1 silencing in GCaMP6m‐MDA‐MB‐231 cells. Data shown are the mean ± SEM of three biological replicates, and statistical analysis was performed using a paired *t*‐test for graph A and multiple *t*‐test comparing NT control to NCS‐1 siRNA for graph B. (C) Gene expression correlation between NCS‐1 and specific plasma membrane Ca^2+^ influx channels using the TCGA breast cancer patient database on the R2: Genomics Analysis and Visualization Platform (http://r2.amc.nl).

Given the ability of NCS‐1 silencing to phenocopy the effects of ORAI1 silencing in MDA‐MB‐231 breast cancer cells, we explored the possibility that NCS‐1 silencing‐mediated suppression of unstimulated Ca^2+^ influx was due to reduced expression of ORAI1. As shown in Fig. 5A, ORAI1 mRNA levels were affected neither by NCS‐1 silencing nor by the expression of its regulators, STIM1 and STIM2. The expression of the major ER Ca^2+^ regulators, IP_3_R and SERCA pumps, were also unaffected by NCS‐1 silencing (Fig. 5B). We also assessed the correlation between NCS‐1 and Ca^2+^ influx channels using TCGA breast cancer patient datasets on the R2 Genomics Visualization Platform as shown in Fig. 5C. Among the assessed genes, the most positively correlated genes with NCS‐1 were TRPV6 (*R* = 0.392), TRPM8 (*R* = 0.340), and ORAI1 (*R* = 0.23), whereas the most negatively correlated genes were ORAI3 (*R* = −0.376) and TRPM7 (*R* = −0.296).

### 3.5 NCS‐1 and ORAI1 silencing enhances percentage of cell death with doxorubicin treatment

**Figure 6:**
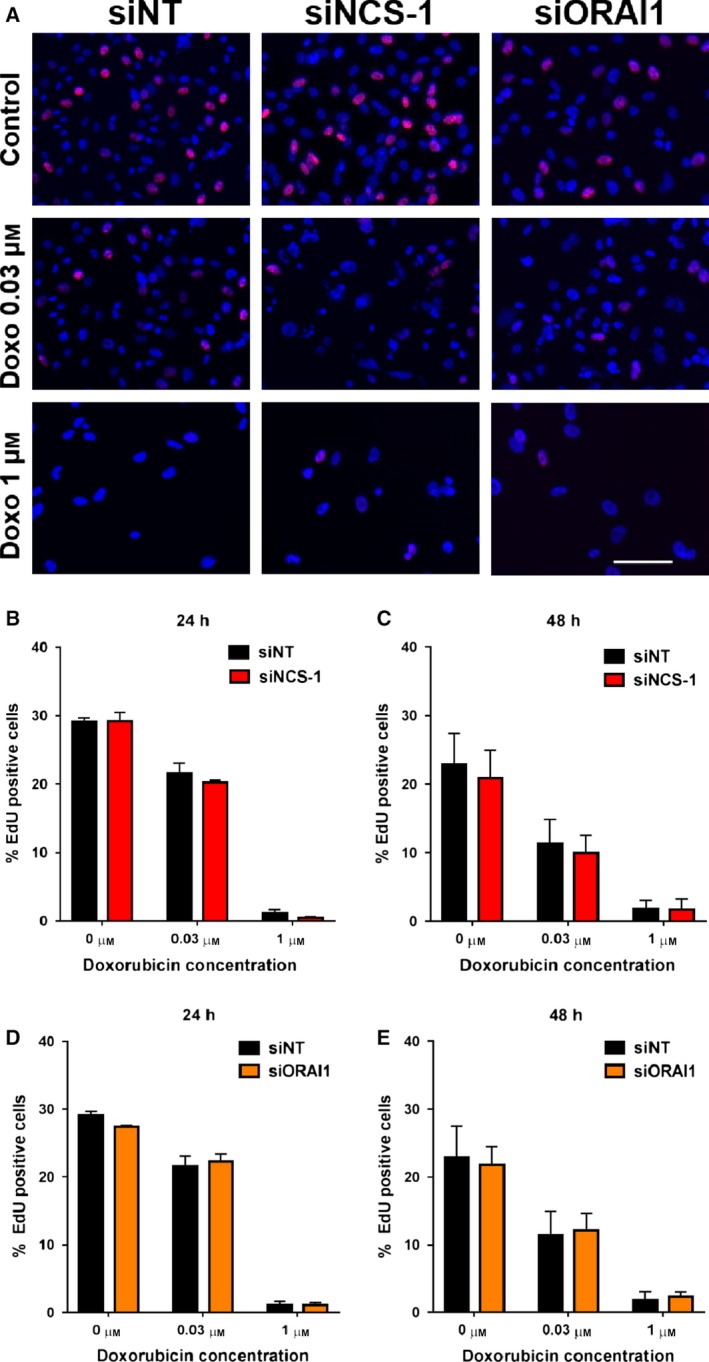
NCS‐1 and ORAI1 silencing does not affect proliferation of GCaMP6m‐MDA‐MB‐231 cells nor promote the antiproliferative effect of doxorubicin. (A) Representative images of NT, NCS‐1, and ORAI1 siRNA‐transfected cells stained with EdU and Hoechst 33342 48 h after doxorubicin treatment (Scale bar = 50 µm). Graphs show the percentage of EdU‐positive cells in NCS‐1 and ORAI1 silenced cells 24 h (B, D) and 48 h (C, E) after doxorubicin treatment. Data shown represent mean ± SEM of four regions in duplicate wells from three independent experiments. Statistical analysis was performed using a repeated‐measures two‐way ANOVA with Bonferroni’s *post‐hoc* test.

**Figure 7:**
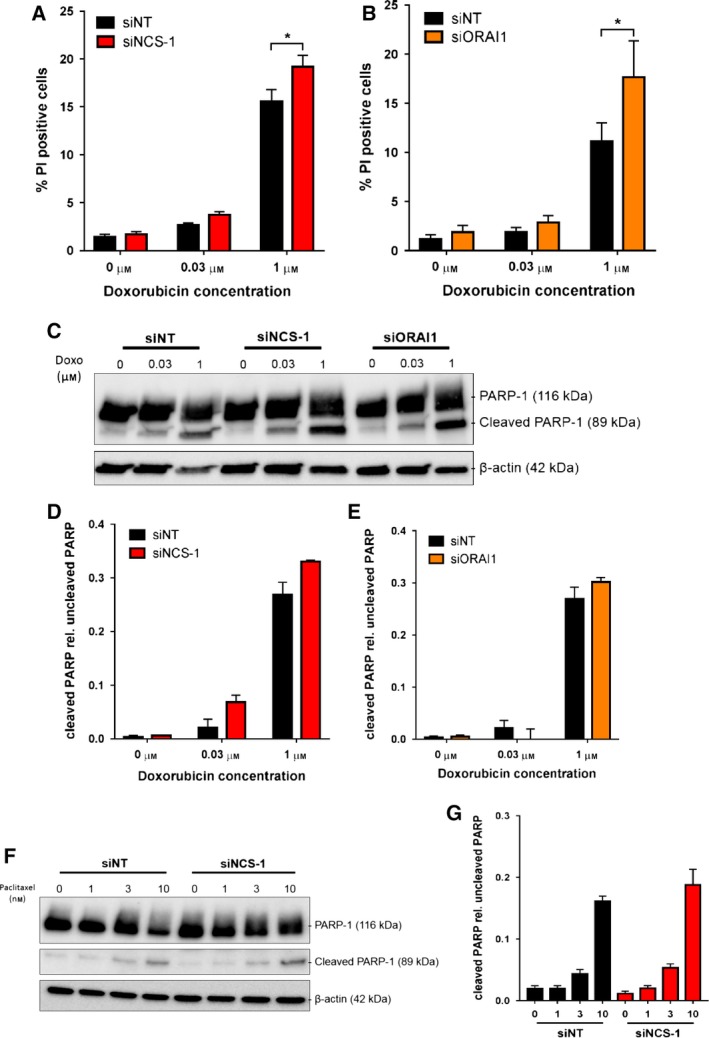
NCS‐1 and ORAI1 silencing promotes nonapoptotic cell death mediated by doxorubicin. Percentage of cell death assessed using PI staining in (A) NCS‐1 siRNA and (B) ORAI1 siRNA‐transfected cells. Data show the mean ± SEM of three independent experiments. (C) Representative immunoblot showing the effect of NCS‐1 and ORAI1 silencing on PARP‐1 cleavage induced by doxorubicin treatment. Bar graphs (D) and (E) show the mean ± SEM of three independent experiments of the ratio of cleaved PARP‐1 to uncleaved PARP‐1 (each band normalized to β‐actin). (F) Representative immunoblot showing the effect of NCS‐1 silencing on paclitaxel‐induced PARP‐1 cleavage. (G) Bar graph shows the mean ± SEM of three independent experiments of the ratio of cleaved PARP‐1 to uncleaved PARP‐1 (normalized to β‐actin). Statistical analysis was performed using a repeated‐measures two‐way ANOVA with Bonferroni’s test. *P < 0.05.

Despite reports of NCS‐1 being associated with therapeutic response to paclitaxel (Moore *et al.*, 2017; Moore *et al.*, 2018), it remains unknown if this is associated with changes in Ca^2+^ signaling in breast cancer cells. Chemosensitization through the suppression of Ca^2+^ influx via inhibition of SOCE has been reported in pancreatic and liver cancer models (Kondratska *et al.*, 2014; Tang *et al.*, 2017). We therefore assessed if ORAI1 and NCS‐1 silencing both augment the effects of doxorubicin, a commonly used chemotherapy in the treatment of basal or TNBCs. We first assessed if silencing of NCS‐1 and ORAI1 promoted the antiproliferative effects of doxorubicin using EdU staining. As shown in Fig. 6A, NCS‐1 and ORAI1 silencing alone did not alter proliferation of MDA‐MB‐231 cells under these conditions. NCS‐1 and ORAI1 silencing also did not augment antiproliferative effects of doxorubicin at 24 h (Fig. 6B,D) or 48 h (Fig. 6C,E). We next assessed the potential of NCS‐1 silencing to promote cell death induced by doxorubicin using PI staining. As shown in Fig. 7A,B, both NCS‐1 and ORAI1 silencing alone did not induce cell death. However, with doxorubicin treatment, NCS‐1 silencing significantly enhanced the percentage of PI‐positive cells induced by doxorubicin (1 µm) treatment (Fig. 7A). This increase in doxorubicin‐ (1 µm)‐induced cell death was phenocopied by ORAI1 silencing (Fig. 7B).

We further investigated if the promotion of cell death with NCS‐1 and ORAI1 silencing is a result of increased apoptotic cell death by assessing PARP‐1 cleavage. As shown in Fig. 7C–E, although doxorubicin treatment resulted in a concentration‐dependent increase in PARP cleavage, both NCS‐1 and ORAI1 silencing did not promote PARP cleavage at any concentration. The lack of the effect of NCS‐1 silencing on promoting apoptotic cell death was also observed with paclitaxel (Fig. 7F,G).

## 4 Discussion

Neuronal calcium sensor‐1 is a recently identified negative prognostic indicator for breast cancer (Moore *et al.*, 2017). In this study, we report for the first time that NCS‐1 is more highly expressed in the basal molecular subtype, a subtype which has a strong overlap with TNBC and is associated with poorer survival rates in breast cancer patients (Prat *et al.*, 2015; Rivenbark *et al.*, 2013; Sorlie *et al.*, 2003). We also observed that higher levels of NCS‐1 predict poorer survival within the basal molecular breast cancer subtype. The association between increased NCS‐1 expression and basal‐like subtypes was also supported in our assessment of the more recently identified TNBC molecular subtypes (Burstein *et al.*, 2015), since higher levels of NCS‐1 was seen in both the BLIA and the BLIS subtypes compared to the mesenchymal (MES) and the luminal androgen receptor (LAR) subtypes. Moreover, a positive correlation between NCS‐1 gene expression and genes typically associated with a basal molecular signature was observed (Prat *et al.*, 2015). Our identification that NCS‐1 is most associated with the basal breast cancer further defines the potential subtype‐specific contribution of NCS‐1 to breast cancer progression.

Despite the recently reported association between NCS‐1 and increased breast cancer invasion and migration (Apasu *et al.*, 2019; Moore *et al.*, 2017), the role of NCS‐1 in Ca^2+^ signaling in breast cancer cells has not been reported. IP_3_‐mediated Ca^2+^ signaling is initiated by a variety of proproliferative and promigratory receptors (Mound *et al.*, 2017; Szatkowski *et al.*, 2010) and could be predicted to be augmented as a consequence of elevated levels of the IP3R‐positive modulator NCS‐1 in basal breast cancers. Given increased activity of IP3Rs with NCS‐1 and the recent report that cardiac cells isolated from NCS‐1 knockout mice exhibit reduced Ca^2+^ transients with ATP stimulation (Nakamura *et al.*, 2011), we hypothesized that NCS‐1 silencing in MDA‐MB‐231 cells would suppress Ca^2+^ mobilization induced by ATP via purinergic receptors and also by trypsin, which activates protease‐activated receptors (Mari *et al.*, 1996). However, there was no major change in IP_3_‐mediated Ca^2+^ signals with NCS‐1 silencing in MDA‐MB‐231 breast cancer cells. Only a modest but statistically significant reduction in the [Ca^2+^]_CYT_ increase induced by 100 nm trypsin was observed. The lack of a major effect on ER Ca^2+^ release after G‐protein‐coupled receptor activation could be explained by a compensatory response, such as an upregulation of components of IP_3_‐mediated Ca^2+^ store release. Analogous to such a change is the upregulation of IP3R1 in mouse embryonic fibroblasts deficient in presenilins (Kasri *et al.*, 2006), proteins that have also been proposed to promote the loss of Ca^2+^ from the ER (Tu *et al.*, 2006). However, our studies found no change in IP3R or SERCA mRNA levels as a consequence of NCS‐1 silencing. These observations suggest that the role and function of NCS‐1 in ER Ca^2+^ release in MDA‐MB‐231 cells are not straightforward. Indeed, studies in neuronal cells reported that NCS‐1 binds to different Ca^2+^ regulators, which are important for the fine‐tuning of Ca^2+^ signals regulating specific processes such as neurite elongation and branching (Hui *et al.*, 2007; Hui *et al.*, 2006; Iketani *et al.*, 2009).There have been no studies assessing the effect of NCS‐1 silencing on Ca^2+^ signaling in MDA‐MB‐231 cells despite two consecutive studies demonstrating a role for NCS‐1 in promoting tumor cell migration and aggressiveness in the same cell line (Apasu *et al.*, 2019; Moore *et al.*, 2017). Based on our studies, it therefore appears that NCS‐1 silencing can modulate cell death and perhaps migration (Apasu *et al.*, 2019; Moore *et al.*, 2017) in MDA‐MB‐231 cells with only a modest or even no effects on activated IP_3_‐mediated Ca^2+^ release.

An alternative explanation is that basal breast cancer cells with elevated NCS‐1 have an upregulation of unstimulated basal Ca^2+^ influx, perhaps to maintain ER Ca^2+^ store levels or via another mechanism. ER Ca^2+^ leak via IP_3_Rs occurs in a variety of cell types (Bittremieux *et al.*, 2016; Boutin *et al.*, 2015). Consistent with this hypothesis, we found that NCS‐1 silencing suppressed basal Ca^2+^ influx in MDA‐MB‐231 cells. One obvious mechanism for such a compensatory pathway is the store‐operated Ca^2+^ channel ORAI1 which is activated by lowered ER Ca^2+^ levels (Brandman *et al.*, 2007; Subedi *et al.*, 2018). ORAI1 is implicated in basal Ca^2+^ influx important for maintaining ER Ca^2+^ homeostasis (Brandman *et al.*, 2007; Zuccolo *et al.*, 2018) in a variety of cell types, although other channels including TRPV6 are also implicated in basal Ca^2+^ influx (Lehen'kyi *et al.*, 2007; Peng *et al.*, 1999; Peters *et al.*, 2012). However, MDA‐MB‐231 cells have negligible (Peters *et al.*, 2012) or undetectable levels of TRPV6 (G.R. Monteith & D. McAndrew, unpublished data), so this compensation is likely to be via the ORAI1 channel in this model. Indeed, the silencing of ORAI1 phenocopied the suppression of unstimulated Ca^2+^ influx by NCS‐1 silencing in our studies. The lack of change in ORAI1, STIM1, and STIM2 mRNA levels with NCS‐1 silencing suggests that nontranscriptional mechanisms such as altered trafficking or activity are involved. It could simply be the case that the reduced loss of Ca^2+^ from the ER with NCS‐1 silencing is detected immediately by STIM1 or STIM2 resulting in less ORAI1 activation in the resting cells and, in turn, reducing the compensatory basal Ca^2+^ influx. Such a mechanism is supported by the lack of effect of NCS‐1 silencing on maximally activated SOCE. Alternatively, ORAI1 can be regulated by phosphoinositides (Calloway *et al.*, 2011; Walsh *et al.*, 2009). NCS‐1 silencing could thus suppress ORAI1‐mediated basal Ca^2+^ influx via this mechanism, as NCS‐1 was shown to regulate phosphoinositide remodeling in PC12 rat adrenal cells (Koizumi *et al.*, 2002). Our studies also do not completely discount the possibility that NCS‐1 regulates other Ca^2+^ influx channels, such as voltage‐gated Ca^2+^ channels or TRP channels as observed in presynaptic neuronal cells (Yan *et al.*, 2014) and PC12 cells (Hui *et al.*, 2006). These mechanisms should be explored in future studies assessing the link between NCS‐1 and Ca^2+^ signaling in cancer cells.

Although exogenous NCS‐1 overexpression could be predicted to promote unstimulated Ca^2+^ influx, since silencing of NCS‐1 inhibited this Ca^2+^ influx pathway, our studies using NCS‐1 overexpressing cells did not show this result. This suggests that NCS‐1 levels in MDA‐MB‐231 may already maximally activate unstimulated Ca^2+^ influx. In this context, Moore *et al*. reported that silencing of NCS‐1 in MDA‐MB‐231 suppressed wound closure but NCS‐1 overexpression in MDA‐MB‐231 cells did not promote wound closure (Moore *et al.*, 2017). Hence, NCS‐1 may already have a maximal contribution to a variety of processes in MDA‐MB‐231 cells at endogenous expression levels. Given the established promigratory role of ORAI1 in MDA‐MB‐231 breast cancer cells (Yang *et al.*, 2009), and our identified potential link between ORAI1‐mediated unstimulated Ca^2+^ influx and NCS‐1, the role of ORAI1 on the effects of NCS‐1 silencing on MDA‐MB‐231 breast cancer cell migration should be assessed in future studies.

Further association between NCS‐1 and ORAI1 was seen in breast cancer samples where a positive correlation was observed between these two genes. NCS‐1 was also significantly positively correlated with the aforementioned channel TRPV6 that is associated with unstimulated Ca^2+^ influx in many cells of epithelial origin (Lehen'kyi *et al.*, 2007; Peters *et al.*, 2012). These data suggest that NCS‐1‐overexpressing breast cancer cells may compensate for enhanced ER Ca^2+^ loss through an upregulation of Ca^2+^ channels involved in unstimulated Ca^2+^ influx. Assessment of the role of TRPM8, which was also positively correlated with NCS‐1, may be challenging given the absence of TRPM8 in many commonly used breast cancer cell lines (Yapa *et al.*, 2018). Alternatively, the positive association between ORAI1 and NCS‐1 may be due to their association with breast cancers of the basal molecular subtype (Azimi *et al.*, 2019). Likewise, lower levels of ORAI3 would indeed be predicted in the breast cancer cells with high levels of NCS‐1 since ORAI3 levels are found to be lower in basal breast cancers (Azimi *et al.*, 2019), which we have shown to have higher levels of NCS‐1.

We also assessed the consequences of silencing NCS‐1 on the effect of doxorubicin, a chemotherapeutic agent used in the treatment of TNBC (Gadi and Davidson, 2017), and whether ORAI1 silencing could phenocopy any effects of NCS‐1 silencing. We identified that both NCS‐1 and ORAI1 silencing enhanced the cell death induced by doxorubicin (1 µm) as determined using PI staining. We further showed that NCS‐1 and ORAI1 silencing did not promote doxorubicin‐induced PARP cleavage. These combined observations suggest that the mode of increased cell death is likely to be necrosis and not apoptosis. We hypothesize that the enhanced doxorubicin‐induced cell death with NCS‐1 silencing is mediated through its regulation of unstimulated basal Ca^2+^ influx. This is supported by our observation that silencing of ORAI1, which mediates basal Ca^2+^ influx in a variety of cells (Brandman *et al.*, 2007; Ross *et al.*, 2013; Subedi *et al.*, 2018), also phenocopied the effects of NCS‐1 silencing on doxorubicin‐induced cell death. Indeed, suppression of basal Ca^2+^ influx increases the effect of other cancer agents, as seen with the promotion of tamoxifen‐induced cell death with TRPV6 silencing in T47D breast cancer cells (Bolanz *et al.*, 2008; Peters *et al.*, 2012).

## 5 Conclusion

This work defines a clear association between NCS‐1 and the basal breast cancer molecular subtype, a subtype with poor prognosis. Our study is the first to identify an association between NCS‐1 and unstimulated basal Ca^2+^ influx in breast cancer cells and comprehensively characterize the role of NCS‐1 in Ca^2+^ homeostasis in breast cancer cells. NCS‐1 silencing also enhanced cell death induced by doxorubicin treatment in MDA‐MB‐231 breast cancer cells. Further studies characterizing the role of NCS‐1 in specific intracellular Ca^2+^ and survival signaling pathways and in other basal breast cancer cell lines are now warranted. These findings will provide new insights into the potential role of NCS‐1 as a modulator of therapeutic responses in basal breast cancers.

## Conflict of interest

GM and SRT hold patents related to ORAI1 in breast cancer.

## Author contributions

AHLB wrote the paper and conducted the experiments, MR performed lentiviral transduction of cells and contributed to the writing of the paper, MJGM performed bioinformatics analysis and contributed to the writing of the paper, SJRT designed experiments and contributed to the writing of the paper, and GRM designed experiments and wrote the paper.
